# Iodine source apportionment in the Malawian diet

**DOI:** 10.1038/srep15251

**Published:** 2015-10-27

**Authors:** M. J. Watts, E. J. M. Joy, S. D. Young, M. R. Broadley, A. D. C. Chilimba, R. S. Gibson, E. W. P. Siyame, A. A. Kalimbira, B. Chilima, E. L. Ander

**Affiliations:** 1 Inorganic Geochemistry, Centre for Environmental Geochemistry, British Geological Survey, Nottingham, NG12 5GG, UK; 2 School of Biosciences, University of Nottingham, Sutton Bonington Campus, Loughborough, LE12 5RD, UK; 3 Ministry of Agriculture, Irrigation and Water Development, Lunyangwa Research Station, PO Box 59, Malawi; 4 University of Otago, PO Box 56, Dunedin, New Zealand; 5 Department of Home Economics and Human Nutrition, Bunda College, Lilongwe University of Agriculture and Natural Resources, PO Box 219, Lilongwe, Malawi; 6 Community Health Sciences Unit, Ministry of Health, Private Bag 65, Lilongwe, Malawi

## Abstract

The aim of this study was to characterise nutritional-I status in Malawi. Dietary-I intakes were assessed using new datasets of crop, fish, salt and water-I concentrations, while I status was assessed for 60 women living on each of calcareous and non-calcareous soils as defined by urinary iodine concentration (UIC). Iodine concentration in staple foods was low, with median concentrations of 0.01 mg kg^−1^ in maize grain, 0.008 mg kg^−1^ in roots and tubers, but 0.155 mg kg^−1^ in leafy vegetables. Freshwater fish is a good source of dietary-I with a median concentration of 0.51 mg kg^−1^. Mean Malawian dietary-Iodine intake from food, excluding salt, was just 7.8 μg d^−1^ compared to an adult requirement of 150 μg d^−1^. Despite low dietary-I intake from food, median UICs were 203 μg L^−1^ with only 12% defined as I deficient whilst 21% exhibited excessive I intake. Iodised salt is likely to be the main source of dietary I intake in Malawi; thus, I nutrition mainly depends on the usage and concentration of I in iodised salt. Drinking water could be a significant source of I in some areas, providing up to 108 μg d^−1^ based on consumption of 2 L d^−1^.

Approximately 2 billion people are estimated to be at risk of iodine deficiency disorders (IDD) worldwide, including 240 million school aged children (SAC)^1^. Whilst I deficiency can affect any age group through impaired thyroid metabolism, the most severe consequences occur during foetal and early childhood development. IDDs can manifest as goitre and impaired cognitive development, with I deficiency during pregnancy and lactation the leading cause of mental retardation in infants^2^,^3^,^4^,^5^. The Recommended Nutrient Intake (RNI) is the dietary intake of a micronutrient that is likely to be adequate for 97.5% of individuals in an age- and sex-specific group and has been set at 120, 150 and 200 μg *capita*^−1^ d^−1^ for children, adults and pregnant women, respectively^6^. When studying the adequacy of nutrient supply at population levels, the Estimated Average Requirement (EAR) is preferred, i.e. 64, 107 and 143 μg  *capita*^−1^ d^−1^, for children, adults and pregnant women, respectively^6^,^7^. Excessive intake of I may cause hyper- or hypo-thyroidism, euthyroid goitre or thyroid autoimmunity^8^,^9^. The Tolerable Upper Limit (TUL) is the level of dietary intake at which no evidence of toxicity is demonstrable^10^ and has been set for adults at 600 and 1,100 μg *capita*^−1^ d^−1^ in the EU and USA, respectively^3^,^11^ while it is recommended that those consuming supplements should avoid a dietary intake >500 μg d^−1^ of I^11^.

The I status of populations can be quantified based on prevalence of goitres and, by this measure, the global incidence of IDD increased by 32% between 1993 and 2003^2^,^6^. Alternatively, a population’s I status may be quantified based on urinary I concentration (UIC). Over 90% of ingested I rapidly appears in the urine, thus urine provides a reliable biomarker of recent I intake and is a common method for assessment of individual status owing to ease and cost effectiveness of collection^12^. A population’s I status is defined as ‘inadequate’ if the median UIC is <100 μg L^−1 ^13. Since 2003, the World Health Organisation (WHO), International Council Control Iodine Deficiency Disorders (ICCIDD) and United Nations Children Fund (UNICEF) have used the median UIC of SAC to update nationally representative data on the I status of the general population as SAC are convenient to reach through school based surveys^13^. A decrease in I-deficient countries from 110 in 1993 to 32 in 2012 has been reported, due mainly to the use of iodised salt^14^. Andersson *et al.* (2012)^1^ calculated 241 million SAC to be at risk of IDD in 2011, which extrapolates to 1.88 billion people globally and this represents a decrease of 6.4% since 2007.

The greatest prevalence, of I deficiency among SAC is 39.3% (58 million) in Africa. In Malawi, 35% of the national population, using extrapolation of UIC among SAC to update previous survey data, still present a UIC of <100 μg L^−1 ^1,15, whilst the 2010 Malawi Demographic and Health Survey^16^ reported that 62% of households were consuming adequately iodised salt, defined as I concentration >15 mg kg^−1^. Often the success of a salt iodisation strategy is limited by cultural and economic factors, where there is a lack of consistent and sustained monitoring of iodised salt^1^. Another complication is that up to 90% of I in iodised salt is lost from production to cooking^17^. This might partly explain why there is only a weak correlation between the use of iodised salt and prevalence of UIC < 100 μg L^−1^ for all countries listed in the Global Scorecard^1^ (R^2^ = 0.13; Fig. 1). However, when I is assimilated into foodstuffs, its bioavailability can be up to 99%^18^.

Iodine deficiency can occur in populations who depend on locally sourced food from regions of low soil-I concentration owing to the parent material and poor retention of I in soil influenced by soil pH, redox potential, texture, soil organic matter (OM), oxides of iron (Fe) and aluminium (Al), clay content and mineralogy^19^. Most soil I is derived from methylated forms which are volatilised from seawater and transferred to the soil-plant system via rainfall or dust deposition^20^. The fixation of I in soil has a major role in controlling I availability to plants^19^,^21^. Transformation of inorganic to organic forms of I plays an important role in I immobilisation, especially in the soil-water system^22^. Anionic/oxyanionic I species (e.g. I^−^, IO_3_^−^) are likely to be fixed rapidly into inert humus-bound forms and/or inorganic colloids (e.g. Fe/Al hydrous oxides), in addition to losses through leaching or volatilisation^23^,^24^,^25^. Loss of I from the soil solution as iodide is more rapid (minutes-hours) than iodate (hours-days)^21^. Whitehead (1984)^26^ reported that only a small proportion of soil-I in humid temperate regions is soluble in water. The relative importance of these processes has not yet been studied directly in tropical soils. The proportion of soil-I that is water soluble and therefore mobile and available for plant uptake varies greatly depending on soil type, e.g. 2% in soils from Pakistan^27^, 10% in soils from the UK and Morocco^28^,^29^ and up to 42% in soils from Argentina^30^. Worldwide, the mean total I concentration in soils is 3.0 mg kg^−1^ and most locally grown foods from areas other than coastal regions will not contain sufficient I to meet dietary requirements^31^,^32^. Resolving I fixation rates in tropical soils would inform management strategies for optimising the supply of these elements to crops and livestock, for example, via irrigation^33^,^34^,^35^,^36^,^37^,^38^, breeding^39^ or livestock salt licks^40^.

Adequacy of dietary element supplies for small population groups can be quantified through direct analysis of composite diets or by matching food consumption data from dietary recall with composition data^41^,^42^,^43^. For larger population groups, food consumption data can be derived from household surveys or Food Agriculture Organisation (FAO) Food Balance Sheets (FBSs)^44^,^45^,^46^,^47^,^48^,^49^. Provided that there are accurate and locally-relevant food composition data, quantifying dietary I intakes at the national level can support the monitoring of population I status and inform public health policies to address deficiency.

Locally generated food composition I data are not available for many countries worldwide, including Malawi. Compared to elements such as Fe, with concentrations in the parts-per-million (mg kg^-1^) range which can be analysed by atomic absorption spectrometry, the I content in food matrices is typically present in the parts-per-billion (μgkg^-1^) range and requires more sophisticated and expensive techniques such as Inductively-Coupled Plasma Mass Spectrometry (ICP-MS)^50^. Also, sample preparative factors such as drying temperature and extraction procedure are more critical for I than for most elements owing to the volatility of I and its instability in acid matrices^51^. In this study, the I concentrations of commonly consumed food crops from a range of soil types in Malawi were determined to create a national composition database. Composition data were matched to FBS items to quantify the national average dietary I supply. This approach was compared to other metrics for estimating I status at a population level, including UIC and salt iodisation measurements. In addition, the effects of food-based and agricultural interventions on dietary I supply were modelled. An evaluation of the prevalence of I deficiency in Malawi using these different approaches and possible intervention strategies are discussed.

## Results

**Figure 2:**
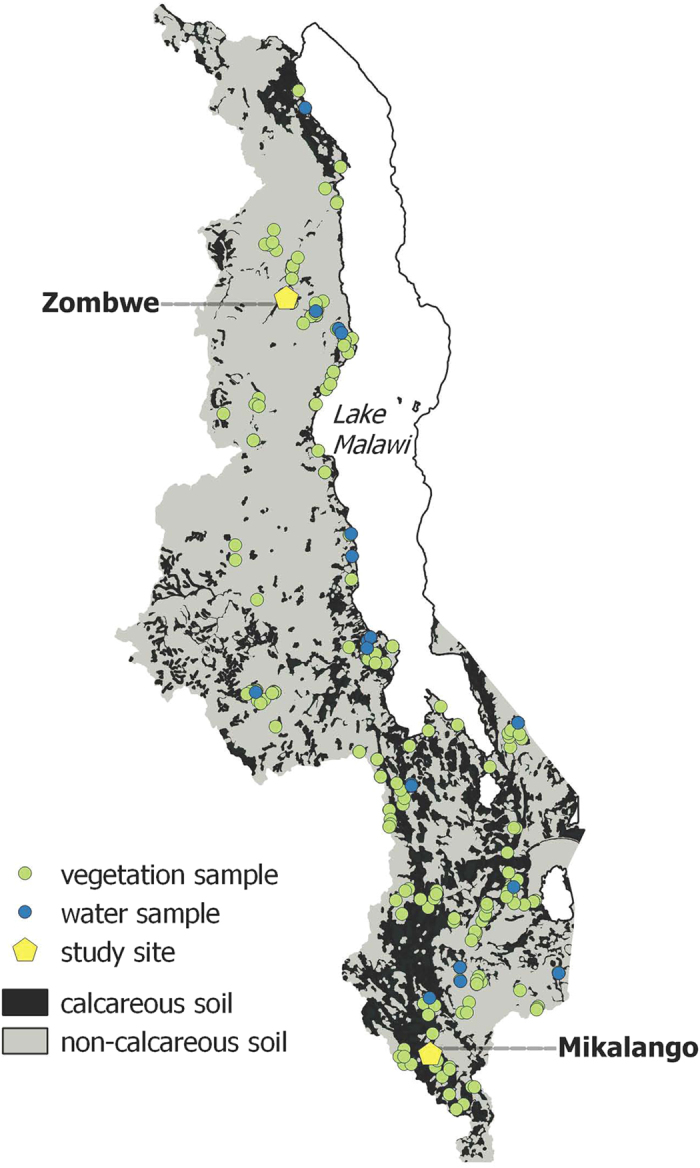
A map of Malawi showing crop and water sampling locations and the two study sites where urine samples were collected (*QGIS Geographic Information System v.2.10.1. Open Source Geospatial Foundation. http://qgis.osgeo.org*)

All data are reported as median (Q1, Q3), unless stated otherwise. Crop and fish data are presented as dry weight (DW). Soil and plant samples were collected across a range of soil types in Malawi with calcareous and non-calcareous characteristics^48^ (Table 1; Fig. 2).

### Soil iodine concentrations

Total soil-I concentration was 2.06 mg kg^−1^ (1.18, 3.35; n = 92), while total I concentrations were similar in calcareous and non-calcareous soils and were, respectively: 1.95 mg kg^−1^ (1.28, 2.65; n = 41) and 2.45 mg kg^−1^ (1.11, 5.07; n = 49; Supplementary Table 1). Concentrations of water-extractable I (with deionised water) were similar between soil types: 0.027 mg kg^−1^ (0.014, 0.042; n = 26) and 0.022 (0.011, 0.032; n = 32) for calcareous and non-calcareous soils, respectively. Total soil-I concentration showed a negative correlation with pH (R^2^ = −0.27) but a positive correlation with organic matter (OM; R^2^ = 0.57). Water-extractable I showed no correlation with pH while the correlation with OM was weaker than for total I (R^2^ = 0.16). Iodine and Se exhibited a positive correlation (R^2^ = 0.73) which may suggest either a common source or simply a reliance on similar soil characteristics for adsorption and retention.

### Food crop and fish iodine concentrations

Maize is the principal staple crop in Malawi supplying >60% of dietary energy intake^55^; the median concentration of I in maize grain was just 0.01 mg kg^−1^ (<0.005, 0.009; n = 82, Supplementary Table 2). The median I concentration was also low in the food group ‘Roots and tubers’, 0.008 mg kg^−1^ (<0.005, 0.015; n = 27) but was greater in leafy vegetables, 0.155 mg kg^−1^ (0.087, 0.250; n = 151). The ranges of I concentrations in the plant tissues analysed were <0.005–1.82, <0.005–0.165, 0.01–0.11, <0.005–0.13, <0.005–0.21, <0.005–0.039, for leaves, grains, pods, seeds, fruits and roots, respectively. Paired soil and plant samples demonstrated no correlation between the I concentration of soil and crops (Table 2; Supplementary Table 3). Fish data from 12 samples had a median I concentration of 0.51 mg kg^−1^ (0.37, 0.87; Supplementary Table 4), despite being sourced from freshwater in Lake Malawi and the Shire River. The greatest concentration of I was found in coconut flesh (5 mg kg^−1^; n = 1).

### Influence of soil factors on food crop iodine concentrations

Previous studies have reported an influence of soil type on elemental concentrations in maize grain and diet composites in Malawi, including Se, Fe and Zn^41^,^43^,^48^,^52^. However, no relationship was observed in this study for paired soil-plant samples between key soil chemical parameters or soil type and plant-I concentration using analysis of variance. For the paired soil-plant samples, soil pH ranged from 4.8 to 8.5, with OM (% LOI) 0.7 to 12.9 (median 2.8; Supplementary Table 3). Water extractable (mobile) soil-I expressed as a percentage of total soil-I (0.2–4.7%) showed no link when correlated with overall plant-I concentrations (R^2^ = 0.018). Soil OM (% LOI) provided a similarly poor relationship with water extractable soil-I (R^2^ = 0.043), whilst soil pH provided a stronger correlation (R^2^ = 0.5476) with a positive, albeit weak soil-I (total) relationship with water extractable soil-I (R^2^ = 0.249). These observations are supported by Bowley (2013)^21^ who reported little uptake of I into grass from soil or from I-129 labelled rainwater (simulated) via the roots, but most likely an increase in plant-I concentrations *via* surface deposition. Weng *et al.* (2009)^53^ reported that soil can provide a low but stable level of mobile soil-I to plants, with enrichment factors in plant tissues increasing from root through to the leaf, demonstrated using I-125.

### Dietary iodine supplies from foods

Food crop and fish I concentrations were matched to mean national dietary supplies of 92 edible food items in 2011^54^. Thus, national mean dietary I supply from foods other than salt was just 7.8 μg *capita*^−1^ d^−1^. Mean supply of I from the food groups ‘Cereals’, ‘Animal products’, ‘Roots and tubers’, ‘Fruits and vegetables’ and ‘Pulses and beans’ were 3.1, 2.6, 1.1, 0.6 and 0.3 μg *capita*^−1^ d^−1^, respectively (Supplementary Table 5). ‘Cereals’, ‘Animal products’ and ‘Roots and tubers’ supplied 40, 33 and 14% of mean national dietary I supplies from foods other than salt, compared to 54, 4 and 18% of mean national dietary energy supplies, respectively^55^.

### Other sources of iodine intake

Salt can be the major source of I intake where iodised salt is consumed. However, water can also provide a significant source of I intake. Iodine data for table/cooking salt samples are limited in the present study (n = 5) with a median I concentration of 53.1 mg kg^−1^ (20.8, 107.1; Supplementary Table 6). In addition, a small number of drinking water samples were collected from hand-dug wells or boreholes, with a median I concentration of 12.6 μg L^−1^ (1.9, 20.9; n = 19; Supplementary Table 7), ranging from 1.0 to 54.2 μg L^−1^.

### Urinary Iodine Concentrations

Urinary Iodine Concentrations (UIC) were measured in a sample of volunteers described in Hurst *et al.* (2013)^41^ in six villages in the Zombwe and Mikalango Extension Planning Areas (EPA). Creatinine-adjusted UIC (μg g^−1^ creatinine) exhibited a median for all 118 volunteers of 203 μg L^−1^ (127, 278 μg L^−1^; n = 118), while median uncorrected UIC was 222 μg L^−1^ (141, 344 μg L^−1^; n = 118; Supplementary Table 8). Table 3 summarises the proportion of individuals that fall into each category of I status from severe deficiency through to severe excess^12^,^56^.

## Discussion

### Soil iodine composition

The median soil-I concentration of 2.06 mg kg^−1^ is below the worldwide mean for regions >50 km from the coastal region of 2.6 mg kg^−1^ reported by Johnson *et al.* (2003a,b)^31^,^32^. Typically, <25% of soil-I is in a soluble form^20^ and in the present study, the median proportion of total soil I that was water-extractable was 1.3% (0.7, 2.0). Soil OM provides a major function in retaining soil-I with humus content influencing the adsorption rate of added iodide and iodate^19^; in the present study, there was a positive correlation between total soil I concentration and OM. In addition, iodide is transformed to organic forms more quickly than iodate^57^; in the present study, there was a negative correlation between total soil I concentration and pH. Subsequently, soils were grouped as ‘high pH, high OM’, ‘high pH, low OM, ‘low-pH, high OM’ or ‘low-pH, low OM’ where ‘high’ and ‘low’ were defined relative to median pH and OM values, respectively. Using this classification, the greatest concentration of total soil I was in the ‘low pH, high OM’ group with a median value of 5.52 mg kg^−1^ (2.82, 8.12) while the lowest concentration was in the ‘high pH, low OM’ group with a median value of 0.95 mg kg^−1^ (0.53, 1.47). Further work is required on I fixation rates as influenced by transformation to organic forms of I and the bacterial role in methylation of I in soil and transfer to the biosphere^24^,^25^. Despite the proportion of water extractable soil-I being greater at soil pH > 8 (3 to 4.7%) than at soil pH < 5 (0.1 to 1%) in this study, calcareous soils have been reported to fix iodate rather than increase bioavailable I for uptake by plants^58^, whereas iodide is a more mobile form of I, favoured by acidic and anaerobic conditions^21^.

### Food crop and fish iodine concentrations

Iodine concentrations in this study were comparable to the limited I data available in the literature for maize grain, fruits and vegetables for Tanzania, West Africa and the UK (Table 1). The data confirm that the majority of fruit, grains and vegetables provide little input to dietary I intake, including from staple foods such as maize. Concentrations of I were greater in fish and leafy vegetables while one sample of coconut flesh had a concentration of 5 mg kg^−1^ (DW); however, coconuts are not widely consumed in Malawi.

No direct correlation was observed between paired soil and plant-I concentrations. Only a weak correlation between water-extractable (mobile) soil-I and plant-I was observed (R^2^ 0.018). The median water-extractable soil-I as a proportion of total soil-I was 1.3%, which was comparable to Pakistani soils (2%)^27^ and significantly lower than 10 and 42% reported for Morocco and Argentina, respectively^29^,^30^.

The limited fish data from the Shire River and Lake Malawi had a median I concentration of 0.51 mg kg^−1^. Thus, a portion of 50 g DW of fish would supply ~25 μg of I to the diet, compared to the adult RNI of 150 μg d^−1^. Iodine concentrations of fish in this study are comparable with the literature. Eckhoff and Maage (1997)^59^ reported wet weight I concentrations 5 to 10 times higher in salt water fish (0.92 mg kg^−1^), compared to fresh water fish in Tanzania, and Haldimann *et al.* (2005)^60^ reported median I concentrations of 1.44 mg kg^−1^ and 0.21 mg kg^−1^ DW in marine and freshwater fish, respectively. Fish and other seafood is recognised as a good source of I and other micronutrients^61^. For example, Maage *et al.* (2008)^62^ demonstrated the use of traditional foods supplemented with marine fish powder to alleviate I deficiency in a cohort of Ghanaian schoolchildren within 14 days.

### Iodine supply from salt

The 2010 Malawi Demographic and Health Survey^16^ reported that 62% of households were using salt iodised at the recommended concentration of ≥15 mg kg^−1^. The median I concentration from the five salt samples collected from a range of grocery stores in the present study was 53.1 mg kg^−1^. However, one sample of packaged salt and one sample of market-bought rock salt had an I concentration >100 mg kg^−1^ with consumption of 5 g d^−1^ providing >500 μg d^−1^ to dietary supplies, risking excessive intake. This finding complements previous studies that question the monitoring of the salt iodisation campaign in Malawi. Kenji *et al.* (2003)^63^ reported I concentrations in supermarket-bought iodised salt well above the recommended range with a mean of 101.6 mg kg^−1^ and in open market vendors a mean of 68.1 mg kg^−1^ and as low as 17.6 mg kg^−1^. The Malawi Micronutrient Survey (2001)^64^ found that only 36% of households were using salt at greater than the recommended concentration of I, whilst the Malawi Demographic and Health Survey^65^ reported successful coverage of 62% of households. Weng *et al.* (2014)^66^ found that 15% of total I was lost during the cooking of biofortified celery, compared to 69% of total I when celery was cooked with iodised salt. A comparison of food preparation processes would be of further interest to study the influence on a range of micronutrients, including I.

### Iodine supply from water

Water-I concentration and water intake are important considerations for assessing overall I intake. For example, in Argentina, a large proportion of drinking water samples from groundwater sources was found to have I concentration >100 μg L^−1^, with potential for significant dietary intake^30^. Consumption of 2 L d^−1^ is often assumed for adults^67^, therefore at the median water-I concentration of 12.6 μg L^−1^ in this study, water may contribute up to 25 μg d^−1^ to daily I intake. The maximum I concentration in drinking water was 54.2 μg L^−1^, potentially contributing up to 108 μg d^−1^ of I. Reimann *et al.* (2003)^68^ reported a wide range of I concentrations in water samples from spring, well and river sources taken along the Rift Valley in Ethiopia (0.31–961 μg L^−1^, n = 138), with a similar median concentration (11 μg L^−1^) to the present study and there is likely to be a wide range of I concentrations in drinking water in Malawi considering the variable surface and groundwater supplies used. Barikmo *et al.* (2011)^69^ reported a median water-I concentration of 108 μg L^−1^ (55–545 μg L^−1^) and a significant positive correlation with UIC measured in Algerian refugee camps. The authors considered the high water-I to be of the cause goitres following excessive I intake from water.

### Urinary Iodine Concentrations

Iodine deficiency as defined by UIC^12^,^56^ was present in 12% (creatinine corrected) of all volunteers from both villages (Table 3). Whilst this sounds promising, it is not an entirely clear picture with greater prevalence of deficiency in Zombwe than Mikalango EPAs. Furthermore, 29% of individuals exhibited moderate excess and 21% severe excess intake of I according to UIC. In addition, a greater proportion of volunteers were I deficient in Zombwe (19%) than Mikalango (5%) which may be due to differences in I consumed from foods, water or salt. Similarly high UIC concentrations in excess of 300 μg L^−1^ were reported in Sudan^70^ and Tanzania^71^ where the consumption of dried fish preserved with salt and consumption of iodised salt occurred together. Out of 47 mainland African countries reported in the 2014 Iodine Global Scorecard^72^,^73^, 14 were classed as having ‘more than adequate’ I and three as ‘excessive’ on the basis of UIC in SAC. Iodine status was defined as ‘insufficient’ in just 10 countries.

### Dietary iodine supplies and deficiencies in Malawi

Data from the range of plant and fish samples in the present study suggest a minimal input of food sources to dietary intake (i.e. <10 μg *capita*^−1^ d^−1^). Previously, Joy *et al.* (2014)^55^ estimated mean national dietary I supplies from foods other than salt to be 36 μg *capita*^−1^ d^−1^ on the basis of FBSs and published composition data. The lower estimate in the present study is due to the use of locally-generated food crop and fish composition data. An individual consuming the mean national I supply from foods, 5 g d^−1^ of salt with I concentration of 53.1 mg kg^−1^ and 2 L d^−1^ of water with I concentration of 12.6 μg L^−1^ would have a total dietary I intake of ~300 μg d^−1^. However, 265 μg of that total intake would be from salt and inadequate dietary I would be extremely likely without consumption of iodised salt.

### Policy implications

Inadequate I supply from foods and variable I supply from water reaffirm the value of the national salt iodisation strategy to alleviating dietary I deficiency. Economic costing can provide impetus to the maintenance of national programmes and the development of cost-effective strategies. Salt iodisation is likely to be highly cost-effective at addressing widespread I deficiency with cost per disability-adjusted life year saved ~US$ 35^74^ while the economic benefit-cost ratio is likely to be 30:1^75^. Salt iodisation has been effective in reducing I deficiency at a global scale with countries assigned as I-deficient falling from 110 to 32 between 1993 and 2012^1^,^76^.

In Malawi, salt iodisation is likely to have improved the I status of the population since its inception in 1995^77^. However, there are challenges remaining. Firstly, coverage of iodised salt is incomplete, with 38% of households not consuming adequately iodised salt in 2010^65^. Secondly, promotion of iodised salt consumption presents a potential conflict with WHO measures to target maximum individual salt intake of 5 g d^−1^ by 2025 to reduce risk of chronic diseases arising from high blood pressure^78^. Mean salt consumption is currently likely to be >5 g *capita*^−1^ d^−1^ in many countries in Africa and Joy *et al.* (2014)^55^ calculated that the risk of dietary I deficiency would increase from 19 to 35% of the population of Africa if there was universal consumption of 5 g *capita*^−1^ d^−1^ of salt. Integration of both strategies to reduce overall salt intake and optimise the level of salt iodisation is essential. Thus, the recommended level of salt iodisation may need to increase by 59% to account for the proposed reduction in salt intake^72^. However, this amplifies the third challenge, being the risk of excessive I intakes, which in Malawi was supported in the present study with I concentration >100 mg kg^−1^ in two of five salt samples and UIC > 300 μg L^−1^ in 21% of adult women from two villages on contrasting soils. Thus, greater monitoring of iodised salt manufacture, packaging, distribution and usage is required. In a similar scenario, Sebotsa *et al.* (2005)^79^ reported that salt iodisation had eliminated IDDs in Lesotho, but a high median UIC of women in lowlands led to a possible I-induced hypothyroidism in vulnerable people.

The present study demonstrates the importance of locally-relevant and accurate composition data for estimating dietary I supplies. Such information can improve the design or adaptation of I intervention strategies appropriate to the region and cultural habits, such as biofortification, salt iodisation or dietary diversification. In addition, simulation models can be improved to predict anticipated effects of food fortification or changes in dietary habits including consumption of salt^50^,^80^,^81^. Combined monitoring of all sources of I intake is required to provide a better prediction of deficiency whilst avoiding excess I intake. Clearly, the risk-benefit is in favour of the fortification of salt with I, but careful monitoring of intake is required to avoid those with the greatest I intakes exceeding the Tolerable Upper Levels^8^.

### Caveats

Creatinine adjustment of UIC has been questioned for use in developing countries because its use may be compromised by the varying intake of protein and water in the diet in these countries, the existence of malnutrition, and through differing rates of hydration^82^,^83^. Correction of UIC can have a considerable influence on values reported, as shown in Table 3. Other methods such as specific gravity^84^ or osmolality^85^ are worthy of further study to ensure reliable dilution correction of UIC to allow for variations in dietary intake, particularly as such importance is placed on UIC measurements to define the success of national campaigns to alleviate IDDs. Ninety percent of dietary I is excreted in the urine and is therefore useful to accurately measure recent I intake. Use of analysis boundaries is of most interest, for example, just below or above the 100 μg L^−1^ (μg g^−1^ creatinine) boundary to define sufficiency, therefore accurate I measurements and corrections for dietary intake or rates of hydration are essential to categorise UIC study groups with confidence. Another limitation of UIC for consideration in the present study is the recommendation by the WHO for the collection of ≥300 casual urine samples from any given population to identify IDD. In addition, prevalence estimates are based on a single urine sample per person, which do not take into account within-subject variation in UIC. Increasingly, investigators are recommending the collection of a second casual sample on at least a subset of the population so that the within-subject variation can be estimated and the distribution of observed UIC adjusted to usual UICs^86^.

Iodine status can easily change between UIC analysis boundaries indicating sufficiency or deficiency of I intake depending on nutrient-nutrient interactions^50^,^87^ and the presence of goitrogens^56^. In order to measure these influences other key micronutrients, such as Se should be measured to correctly assess the risk of thyroid dysfunction affected by nutrient-nutrient interactions^50^, for example, combined deficiencies of I + Se^88^ or Fe + I, Se + I and vitamin A + I^87^. In addition, the complex interaction from inhibiting compounds in foods, such as goitrogens for I, need further investigation.

An additional consideration for the water-I concentrations could include methods of food preparation. For example, partial fermentation by immersion in water is used for processing cassava to remove the goitrogen, linamarin, a cyanoglucoside as well as refined maize flour (65% extraction) known in Malawi as white ufa. After soaking, the cassava and maize flour are dried via exposure to the sun. Both the practices of soaking and drying may impact negatively on the I concentrations of these processed foods. The quantification of goitrogens, such as thiocyanates, flavonoids or pyridines in key foodstuffs, including cassava, is essential to understand the utilisation of I in the thyroid. Goitrogens in drinking water are an important consideration, for example, bacterial contamination can inhibit the degradation of organic pollutants, high concentrations of calcium (Ca) or magnesium (Mg) salts have been implicated, as well as polyaromatic hydrocarbons and other organic compounds often associated with effluent from coal fields^89^.

The low supply of I from foods other than salt (i.e. <10 μg capita^−1^ d^−1^) was estimated from FBS food supply data matched with locally-generated composition data. However, there are inherent weaknesses in FBS data which have been discussed extensively elsewhere^55^,^90^. Compared to FBS data, mean national fish consumption was ~7-fold greater in the 2010–2011 Third Integrated Household Survey of Malawi^16^. Thus, I supply from fish may be under-estimated in the present study.

## Methods

### Sample collection and analysis

Plant and soil samples were collected from farmers’ fields and markets from 2012 to 2014, generally at harvest time (May to July). Plant samples were brushed and washed with distilled water to remove soil and dust particles before being oven-dried at <40 °C. Where possible, soil samples were collected close to where the plant samples were collected, although this could not be done for plant samples bought at markets. Soil samples were air-dried, crushed and sieved to 2 mm. Both plant and soil samples were further ground to <40 μm in an agate ball mill. Fish samples were obtained from markets, representative of typical consumption for the localities. The fish samples were typically fresh water fish from Lake Malawi or the Shire River, often dried prior to purchase and then deboned. Salt samples labelled as iodised salt were collected from a variety of small grocery stores across Malawi. Plant and fish samples were passed through a food blender prior to milling, as appropriate. The analysis of I in soil, plant and fish materials follows the method employed in Watts and Mitchell (2009)^91^ and Watts *et al.* (2010)^30^ for 0.25 g of soil, for which soil-I was extracted using 5% tetramethylammonium hydroxide (TMAH) at 70 °C in a drying oven. Plant and fish samples required a more robust extraction procedure, using microwave heating (MARS Xpress, CEM), which combined heating and pressure to improve the extraction of I within a closed vessel system. Salt samples were dissolved in 1% TMAH, with subsequent analysis of I for all samples carried out by inductively coupled plasma mass spectrometry (ICP-MS; Agilent 7500cx), with the collision cell in no-gas mode. An ASXpress sample introduction loop (CETAC) was used to minimise the volume of sample (500 μL) presented to the ICP-MS and therefore improve on the washout time compared to previously reported iodine analyses by the author^91^ to a limit of detection (LOD) of 0.25 μg L^−1^ (3SD blanks), or 0.01 mg kg^−1^ in the solid. Measurements below the LOD were attributed a concentration of half the LOD. Reference material values for a range of soil and vegetation matrices were comparable with values reported in Watts and Mitchell (2009)^91^.

Soil pH was measured in water using 10 g soil and 25 mL water. Organic matter content was determined as loss on ignition (LOI) at 450 °C for 1 g of soil. Multi-elemental composition data (other than iodine) for maize samples collected in 2011 have been previously reported^92^ and for all other crops collected between 2011 and 2014 in Joy *et al.* (2015)^48^. Urine samples were collected as described by Hurst *et al.* (2013)^41^. Group sizes were sufficiently large enough (>30) to assess UIC status on a median UIC, rather than on a single individual. Group data of >30 people allows for considerable day-to-day variation in urinary I excretion, often effected by varying degrees of hydration, malnutrition or diverse dietary intake^8^,^13^,^93^. Frozen urine samples were thawed at room temperature and shaken gently to homogenise before three ×1 mL aliquots were dispensed for creatinine adjustment, I analysis in a TMAH matrix by ICP-MS (Thermo X-Series 2, ThermoFisher) using a ^185^Re internal standard and multi-element analysis in 2% nitric acid, with the latter reported in Hurst *et al.* (2013)^41^. Certified Reference Material, Seronorm™ provided an accuracy of 85% (n = 8; 260 ± 3 μg L^−1^).

### Assessing the influence of soil factors on the mineral composition of plants

Previous studies have demonstrated the potential influence of soil type on maize grain and diet composite Se, Fe and Zn concentrations^52^. Therefore, plant and soil sample locations were matched to FAO soil classifications available at a national scale^94^ using the ‘spatial join’ function in ArcGIS (v. 9.3, ESRI, Redlands, CA, USA). The FAO soil classes were assigned to two groups: ‘calcareous’ (Calcaric and Eutric classes) and ‘non-calcareous’ (all other classes). Leptosols were not assigned because they cannot be classified on this basis. Using this classification, 69% of the total land area in Malawi is non-calcareous, 26% is calcareous and the remainder is unassigned. The validity of the calcareous/non-calcareous groupings was tested by comparing their soil Ca concentrations and pH, which confirmed that the term ‘calcareous’ indicated the presence of calcium carbonate in the soil and pH > 6.5^48^. Soil type and sampling locations are shown in Fig. 2.

Availability of food composition I data in Africa for comparison of this dataset was limited. Therefore, additional datasets were used to supplement the African surveys, comprising: food-iodine composition data from the UK Department of Health^95^, a specific study in Tanzania^58^, broad survey of West Africa^96^ and a UK Food Standards Agency survey^97^.

The influence of calcareous/non-calcareous soil types on maize grain and ‘leafy vegetables’ I concentrations was assessed using Analysis of Variance (ANOVA, GenStat v.16, VSN International, Hemel Hempstead, UK). A log_10_ transformation was used to reduce skew in the data. The influence of soil type was assessed for maize grain and leafy vegetable as these categories had adequate sample numbers and represent different plant tissues.

## Additional Information

**How to cite this article**: Watts, M. J. *et al.* Iodine source apportionment in the Malawian diet. *Sci. Rep.* **5**, 15251; doi: 10.1038/srep15251 (2015).

## Supplementary Material

Supplementary Information

## Figures and Tables

**Figure 1:**
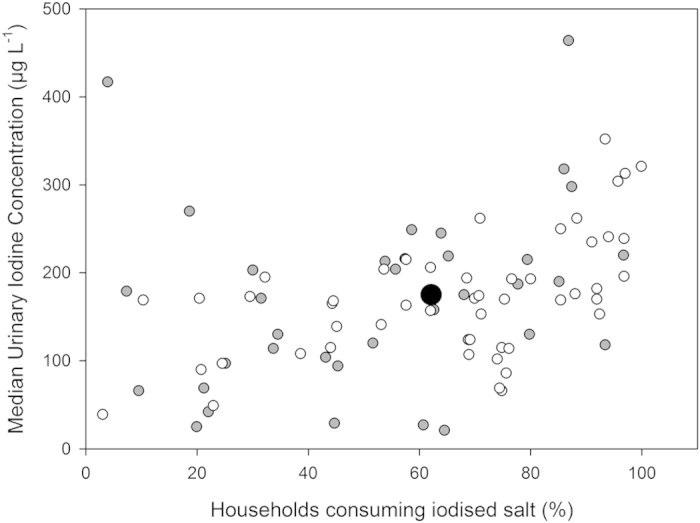
Median UIC as a function of the proportion (%) of households consuming adequately iodised salt. Country data is from the Global Iodine Scorecard 2014. Red squares indicate countries in Africa, black dots are other continents.

**Table 1:**
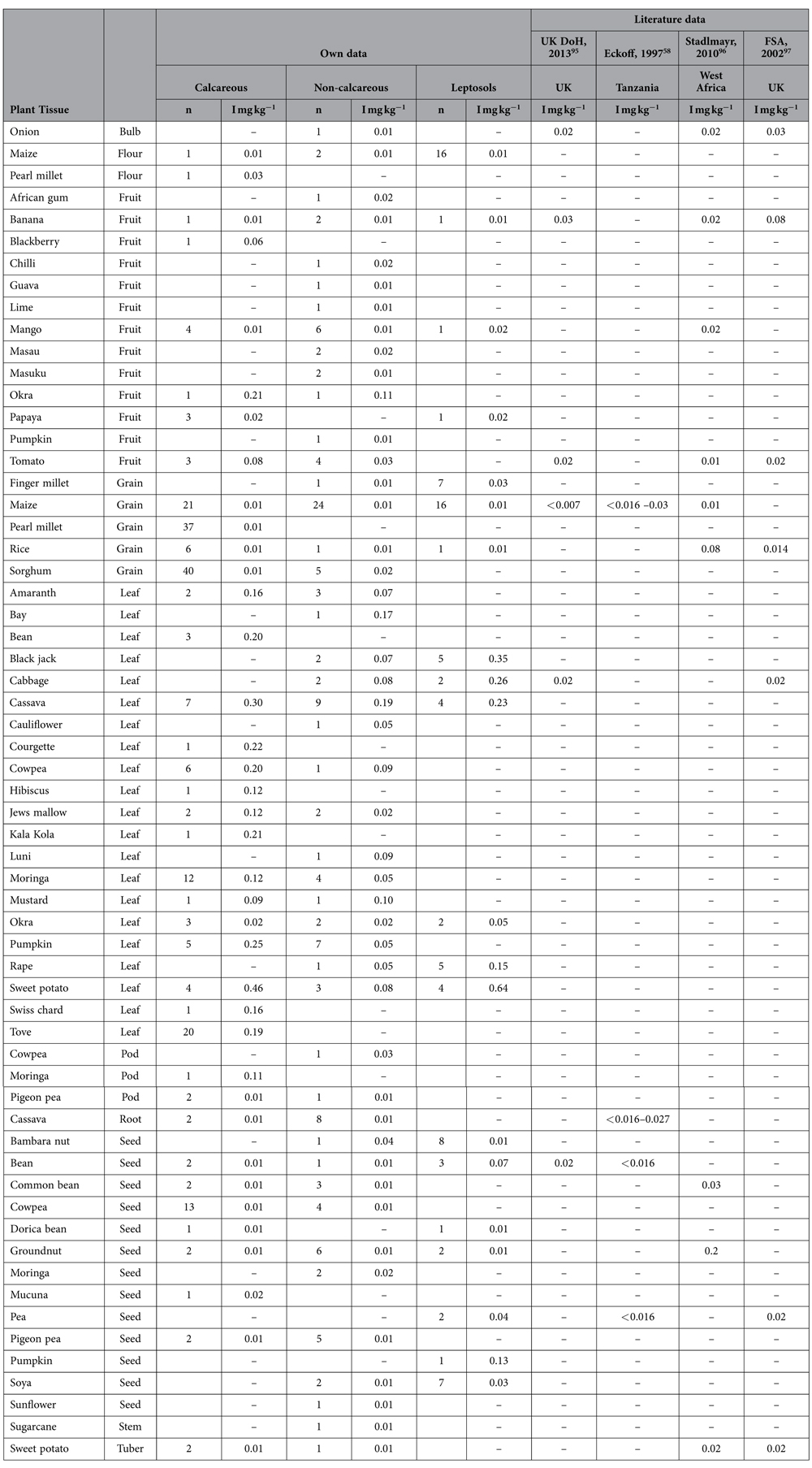
Summary iodine concentrations for paired soil and plants, by plant type and soil classification, compared with available literature data (mg kg^−1^, dry-weight, edible portion).

**Table 2:** Correlation for paired soil and plant iodine concentrations (dry weight − DW).

Plant group	Median iodineconcentration(mg kg^−1^)	SD	CorrelationR^2^
Fruit	0.02	0.02	0.0110
Grain	0.01	0.03	0.0008
Leafy vegetables	0.17	0.24	0.0194
Pods	0.02	0.05	0.0031
Root	0.01	0.01	0.1704
Seeds	0.01	0.03	0.0045

**Table 3:** Proportion (%) of volunteers from Zombwe (n = 59) and Mikalango (n = 59) villages within each sub-group for iodine status as defined by urinary iodine concentrations (UIC) either corrected or uncorrected for creatinine.

Iodine status	UIC	Zombwe		Mikalango		Combined	
	μg L^−1^	Corrected	Uncorr.	Corrected	Uncorr.	Corrected	Uncorr.
deficient	<100	19	29	5	2	12	15
sufficient	100–200	37	34	39	22	38	28
moderate excess	200–300	24	17	34	31	29	24
severe excess	>300	20	20	22	46	21	33
